# Normothermic machine perfusion *versus* static cold storage for liver transplantation: a systematic review and meta-analysis

**DOI:** 10.1590/acb415326

**Published:** 2026-09-11

**Authors:** Miguel Lucas Silva Valente, Angela Claudia Paixão Soares de Magalhães, Hercules Magalhães Olivense do Carmo, Donizete Dalto, Bryan de Sousa Dorea, Marina Marcon Zamban

**Affiliations:** 1 Universidade Federal do Acre – Rio Branco (AC), Brazil.; 2 Fundação Hospital Estadual do Acre – Rio Branco (AC), Brazil.; 3 Universidade Luterana do Brasil – Canoas (RS), Brazil.

**Keywords:** Liver Transplantation, Organ Preservation, Perfusion, Reperfusion Injury

## Abstract

**Purpose:**

To compare the effectiveness of normothermic machine perfusion (NMP) with static cold storage (SCS) in preserving transplantable livers, focusing on early postoperative complications.

**Methods:**

A PRISMA 2020–compliant search of PubMed, Embase, Scopus, Cochrane Library and Web of Science identified comparative studies of NMP versus SCS. Five studies met the criteria (two randomized controlled trials, and three observational cohorts; 630 recipients). Random-effects meta-analysis was performed in RStudio, reporting risk ratio (RR), 95% confidence interval (95%CI), p-value and heterogeneity (I2).

**Results:**

NMP significantly lowered the incidence of composite early postoperative complications versus SCS (RR = 0.60; 95%CI 0.47–0.75; *p* < 0.0001), corresponding to a 40% relative risk reduction. Between-study heterogeneity was negligible (I2 = 0 %). Subgroup analyses revealed no significant effect modification by study design (*p* = 0.75), donation after circulatory death proportion (*p* = 0.91), preservation duration (*p* = 0.51), donor age (*p* = 0.59) or perfusion technique (*p* = 0.37).

**Conclusion:**

NMP demonstrates clinically superior graft preservation compared with SCS, consistently reducing early complications after liver transplantation. Adoption of NMP may expand the use of marginal organs and improve post-transplant outcomes.

## Introduction

Liver transplantation remains the definitive treatment for end-stage liver disease. Nevertheless, the persistent scarcity of suitable grafts restricts its wider application. Static cold storage (SCS) is the historical preservation standard, yet it fails to avert ischemia-reperfusion injury—particularly in marginal grafts—, thereby contributing to early allograft dysfunction and ischemic‐type biliary lesions^1,2^.

Over the past decade, normothermic machine perfusion (NMP) has emerged as a promising strategy to overcome these limitations by maintaining the liver in a metabolically active state *ex vivo*. Randomized trials and real-world series demonstrate that NMP lowers hepatocellular injury markers, early graft dysfunction and ischemic cholangiopathy when compared with SCS, especially for extended-criteria donors and donation after circulatory death (DCD) grafts^3-8^.

Nonetheless, several NMP aspects remain under investigation, including optimal perfusion duration, the impact of combined or sequential strategies and the influence of perfusion parameters (oxygen tension and perfusate composition) on mitochondrial function^9-13^. Experimental data reveal that delayed processing of biopsies after NMP—under hypothermic conditions exceeding four hours—impairs assessment of mitochondrial respiration, underscoring the need for standardized viability protocols^14^.

Finally, cost-effectiveness and logistic feasibility are pivotal for widespread adoption. Although NMP entails higher upfront expenditure, economic modelling suggests that its incorporation may be justified owing to reduced graft discard rates and improved long-term outcomes—considerations highly relevant to contemporary transplant practice^15^.

## Methods

### Eligibility criteria

Randomized controlled trials (RCTs) and observational cohorts comparing NMP with SCS in adult liver transplantation were eligible. Pre-clinical studies, case series, single-case reports and narrative reviews were excluded.

### Information sources and search strategy

A comprehensive search of MEDLINE, Embase, Scopus and Web of Science (inception to January 4, 2025) was performed. Reference lists of relevant systematic reviews were screened manually for additional records.

### Study selection

**Figure 1:**
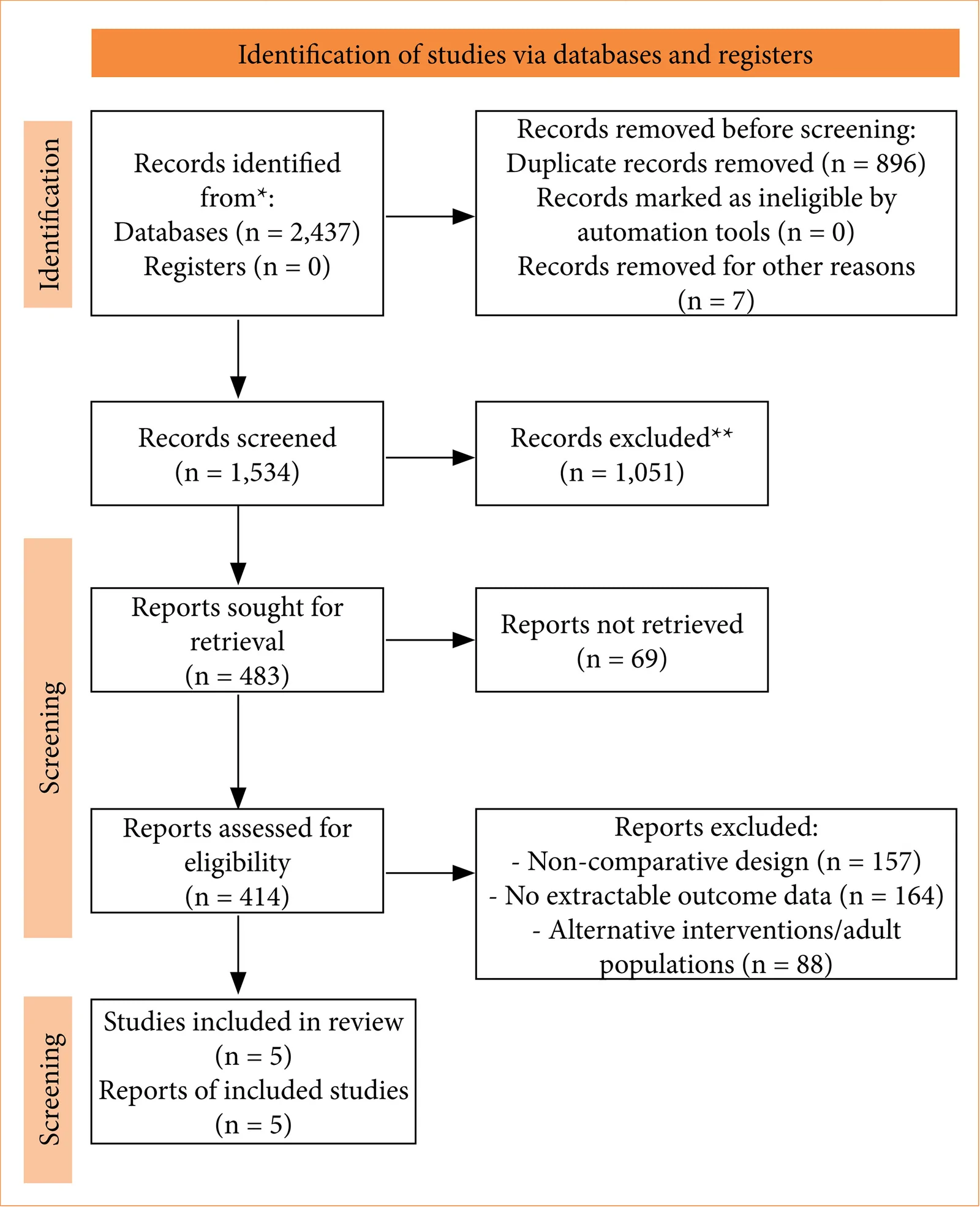
PRISMA flow diagram.

All records were imported into reference-management software. Two reviewers independently screened titles/abstracts and full texts; disagreements were resolved by consensus or third-party adjudication (Fig. 1).

*Records identified from PubMed (n = 820); Embase (n = 710); Cochrane CENTRAL (n = 460); Web of Science (n = 447); **records marked as ineligible by automation tools (n = 0). Source: Elaborated by the authors.

### Data extraction

Two reviewers independently extracted study characteristics, participant details, interventions, and outcomes. Corresponding authors were contacted for clarification or missing data when required.

### Risk-of-bias assessment

RCTs were appraised with Cochrane RoB 2.0, and observational studies with ROBINS-I. Discrepancies were resolved by discussion.

### Data synthesis and statistical analysis

Dichotomous outcomes were pooled using the Mantel–Haenszel method under a random-effects model. Heterogeneity was quantified via I^2^ and τ^2^. Pre-specified subgroup analyses and meta-regressions explored potential effect modifiers. Two-sided *p* < 0.05 denoted statistical significance. Analyses were conducted in R 4.2.2 (metafor package).

## Results

### Overall characteristics and pooled analysis

**Figure 2:**
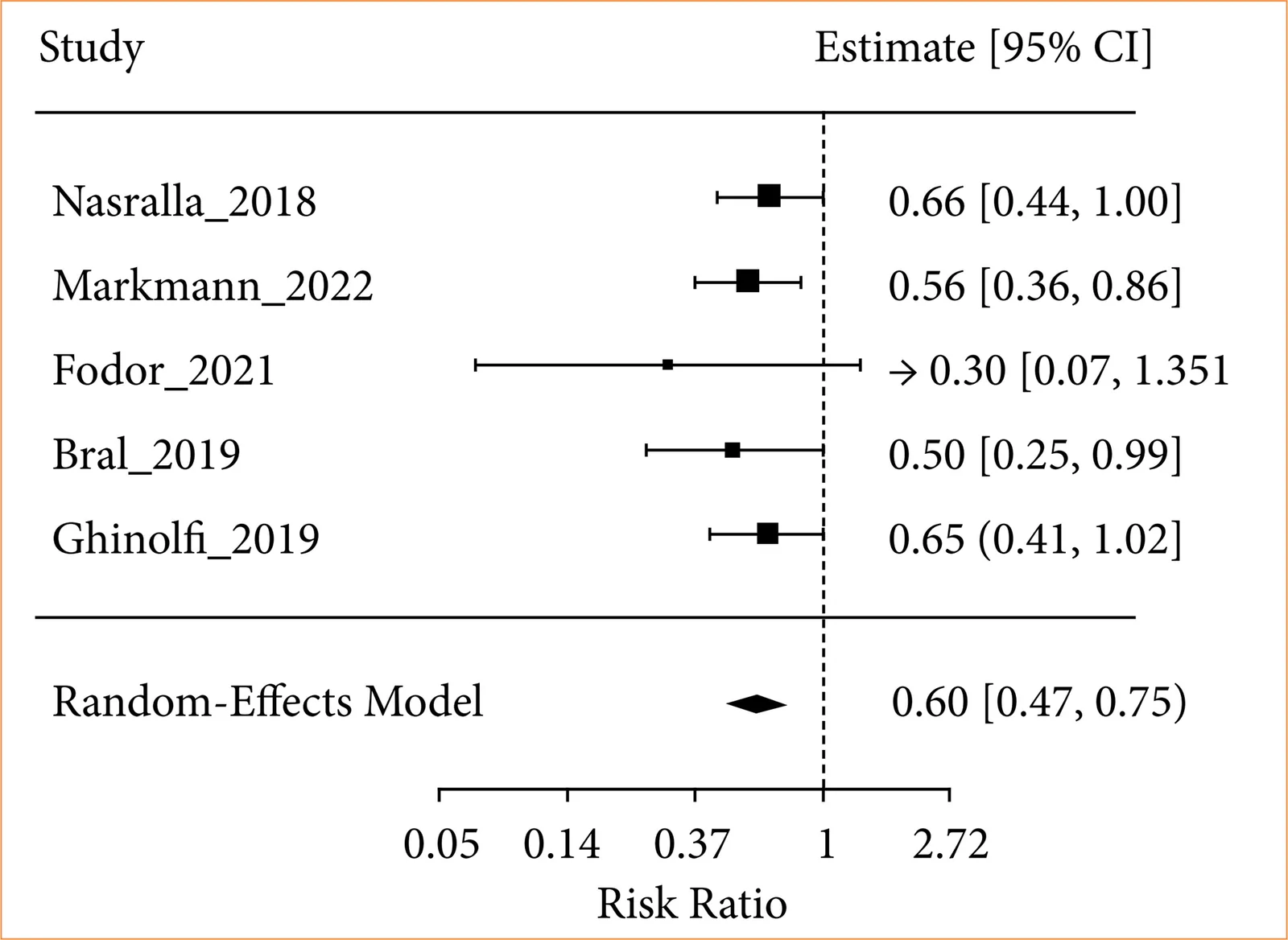
Forest plot of the primary meta-analysis.

Five studies encompassing 630 recipients (318 NMP, 312 SCS) met the inclusion criteria. NMP reduced adverse events by ≈ 40 % (risk ratio [RR] = 0.60; 95% confidence interval [95%CI] 0.47–0.75; *p* < 0.0001) (Fig. 2 and Table 1).

**Table 1:** Characteristics of the included studies.

Study	Design	NMP (n)	SCS (n)	DCD (%)	Mean donor age (y)	Preservation time (h)	NMP modality
Nasralla et al.^4^	RCT	121	101	20	54	11.5	Early
Fodor et al.^6^	Observational	59	59	27.1	53	21.0	Back-to-base
Bral et al.^17^	Observational	11	11	15.4	49	15.0	Back-to-base
Markmann et al.^18^	RCT	153	147	36.6	51	10.2	Early
Ghinolfi et al.^19^	RCT	10	10	40	74	13.2	Early

RCT: randomized controlled trial; NMP: normothermic machine perfusion; SCS: static cold storage; DCD: donation after circulatory death.

Source: Elaborated by the authors.

95%CI: 95% confidence interval. Source: Elaborated by the authors.

Heterogeneity was negligible (I^2^ = 0%; τ^2^ = 0), indicating a consistent treatment effect.

### Subgroup analyses

**Figure 3:**
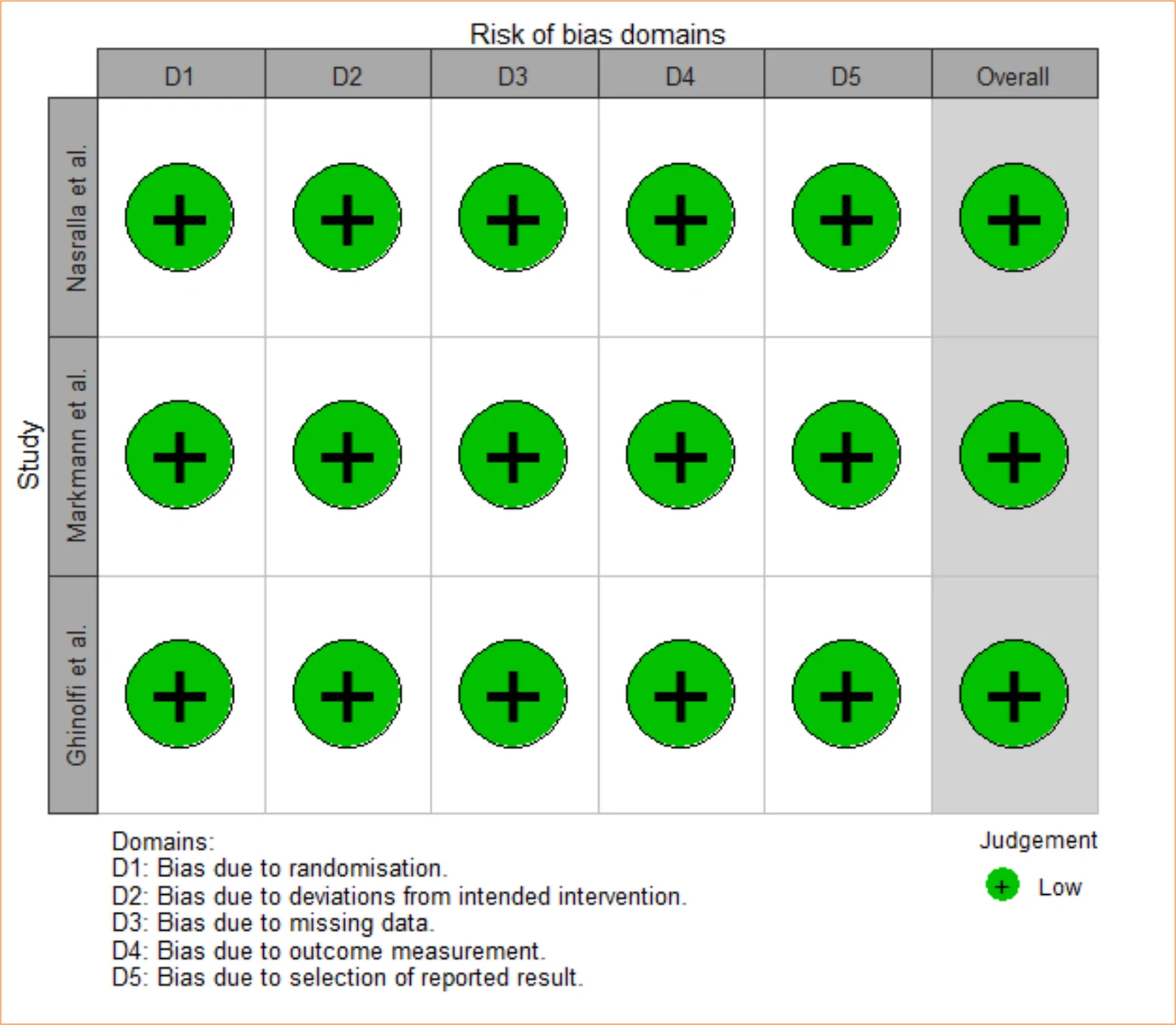
Bubble plots illustrating meta-regressions.

Source: Elaborated by the authors.

Effect estimates were comparable between RCTs and observational cohorts, albeit with wider confidence intervals for cohorts. Continuous meta-regression identified no significant influence of DCD proportion, preservation time, donor age, NMP modality (in-transit *versus* back-to-base) or proportion of chronic indications (Table 2 and Fig. 3).

**Table 2:** Subgroup and meta-regression summary.

Subgroup variable	QM (df)	p-value	Interpretation
Study design	1.2165	0.7491	No significant difference between RCT and observational
DCD proportion (%)	0.0134	0.9079	No significant interaction
Preservation time (h)	0.4355	0.5093	No significant association
Donor age (years)	0.2954	0.5868	No significant association
NMP modality	0.7951	0.3726	Non-significant trend favoring early NMP
Chronic indications (%)	0.0026	0.9591	No significant interaction

DCD: donation after circulatory death; NMP: normothermic machine perfusion; RCT: randomized controlled trial. Source: Elaborated by the authors.

### Sensitivity analysis

**Figure 4:**
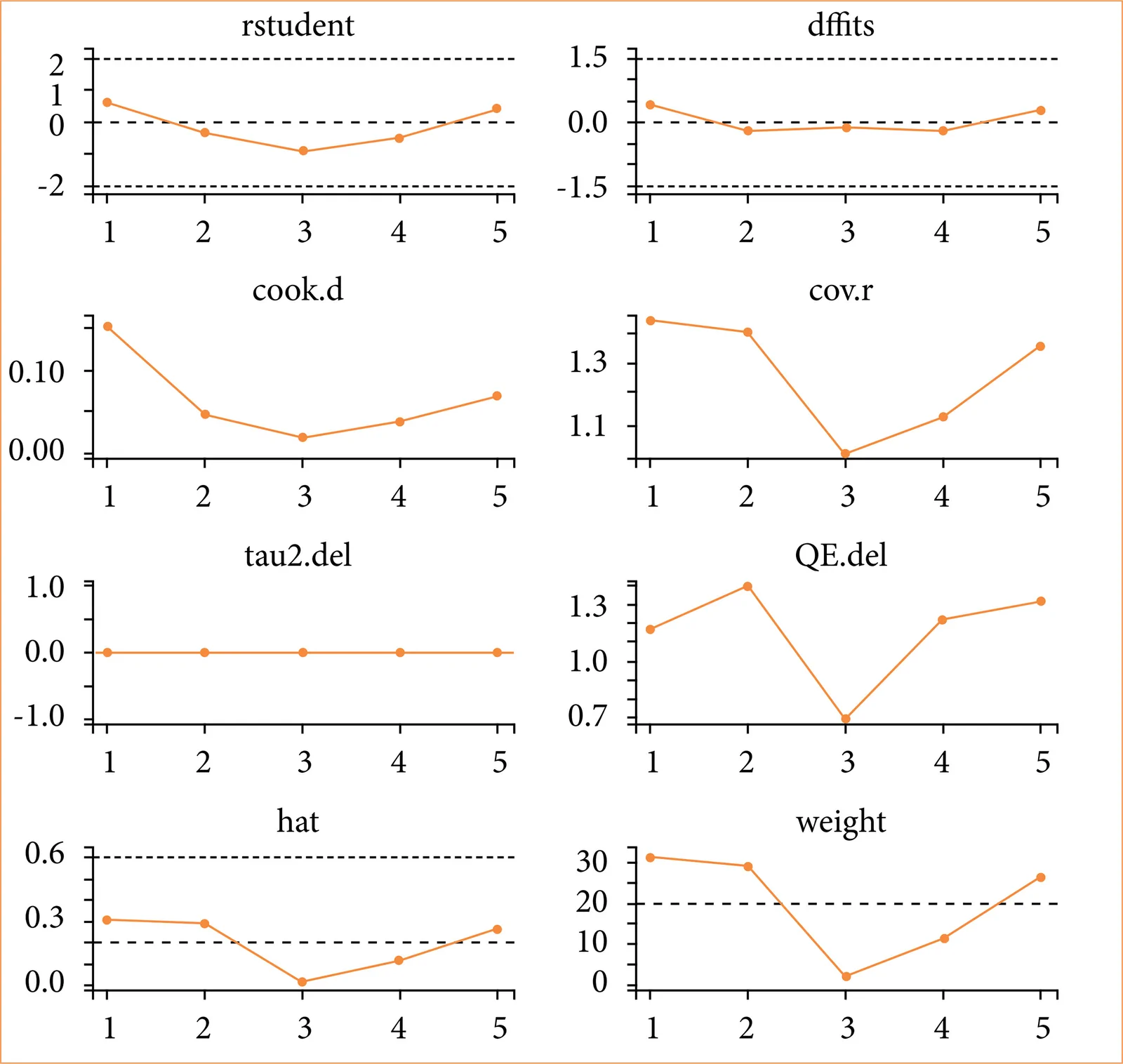
Leave-one-out influence analysis.

Source: Elaborated by the authors.

Leave-one-out analysis confirmed the robustness of the pooled effect; no individual study exerted disproportionate influence (Fig. 4).

### Publication bias

**Figure 5:**
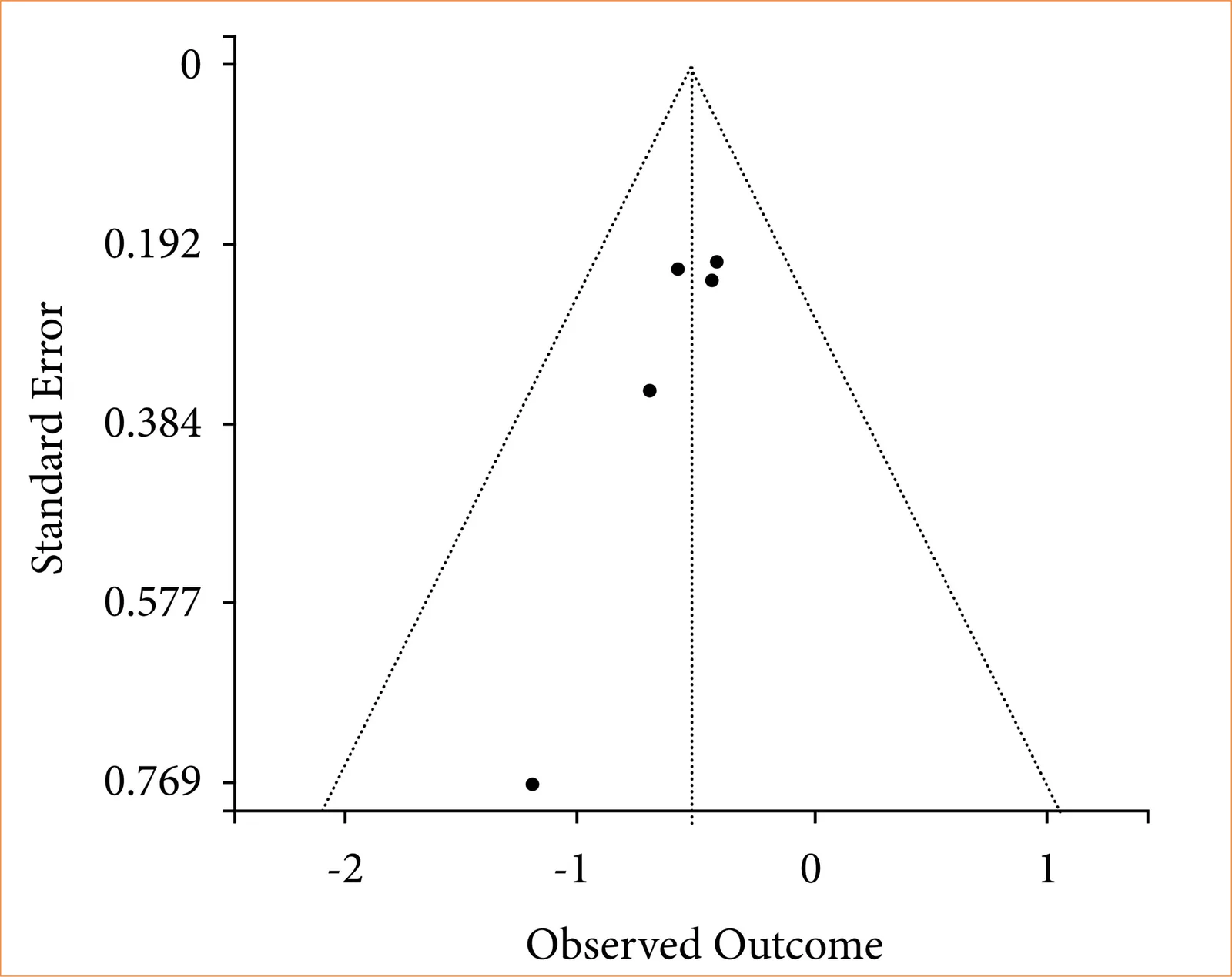
Funnel plot assessing publication bias.

Source: Elaborated by the authors.

The funnel plot was symmetrical, and Egger’s test was non-significant (*p* = 0.2854), suggesting a low likelihood of publication bias (Fig. 5).

## Discussion

This synthesis reinforces that NMP significantly enhances graft viability, reducing early dysfunction and ischemic biliary injury relative to SCS^6,8,16,17^. Benefit remained consistent across donor age strata, preservation times and varying DCD proportions.

Normothermic preservation likely preserves mitochondrial integrity and hepatic energetic homeostasis—critical determinants of post-transplant recovery^9-13^. Experimental observations stress the need for standardized protocols when assessing mitochondrial respiration, as biopsy-processing delays can obscure true graft viability^14^.

From an economic standpoint, modelling studies indicate that, despite higher upfront costs, NMP may be cost-effective by decreasing organ discard and improving long-term outcomes. Logistic hurdles—device availability and staff training—persist but are steadily being overcome in high-volume centers.

### Limitations

The restricted number of studies, methodological heterogeneity among observational cohorts, and variability in outcome definitions limit generalizability. Large, multicenter RCTs with extended follow-up are warranted to assess survival impact and real-world cost implications across diverse healthcare systems.

## Conclusion

NMP markedly reduces adverse events compared with SCS and represents a promising strategy to expand the donor pool and optimize graft function. Current evidence supports considering NMP the new standard for hepatic preservation, particularly for marginal or DCD grafts. Future research should address long-term outcomes, protocol standardization and economic-operational analyses.

## Data Availability

All data are presented in the article.
